# Association between radiation volume and breast density for skin toxicity and breast edema after radiotherapy in breast conserving therapy of breast cancer

**DOI:** 10.1186/s13014-026-02833-w

**Published:** 2026-03-28

**Authors:** Mathias Sonnhoff, Melsa Oyur, Jan-Nicklas Becker, Robert Michael Hermann, Cedric Oliver Carl, Mirko Nitsche, Hans Christiansen, Robert Maximilian Blach

**Affiliations:** 1 Department of Radiotherapy, Hannover Medical School, Carl-Neuberg- Straße 1, 30625 Hannover, Germany; 2 IMAGINE-Niedersachsen, Carl-Neuberg-Str. 1, 30625 Hannover, Germany; 3 Radiotherapy NORD Bremen and Westerstede, Bremen, Germany; 4 Department of Radiation Oncology, University Medical Center Schleswig-Holstein, Kiel, Germany

## Abstract

**Background:**

Despite the favorable toxicity profiles of whole breast radiotherapy, there are subgroups that tend to experience high toxicity. This study examines anatomical and physiological characteristics that can predict the induction of toxicity for skin and edema.

**Methods:**

In the present cohort, we examined 387 patients who received only radiation therapy to the breast, did not receive chemotherapy, and whose planning data could be fully constructed for evaluation. Metric parameters of the therapy that correlate directly with physiological/anatomical characteristics, such as the irradiated PTV and the boost volume, were evaluated. We also measured breast gland density in a defined reference structure (surrogate Parameter). We compared the parameters collected between patients who experienced grade II toxicity after therapy and patients who experienced grade I or less toxicity from the therapy.

**Results:**

There was a significant correlation between volume and grade II toxicity for both skin toxicity and edema formation. There was also a higher risk of grade II for the surrogate marker in patients with a HU of less than − 59. The multivariable model for the parameters confirms the volume of the PTV and the boost as risk factors, but not the HU of less than 59.

**Conclusion:**

There is evidence of a correlation between breast volume, the density of breast tissue and the resulting toxicity of adjuvant radiotherapy in breast cancer. Multivariate analyses were not statistically significant in the retrospective cohort study, and further research is required. Taking these factors into account could further improve treatment tolerance.

**Supplementary Information:**

The online version contains supplementary material available at 10.1186/s13014-026-02833-w.

## Introduction

The efficacy and toxicity profiles of whole breast irradiation (RT), the standard treatment after breast-conserving therapy in curative intention for localized breast cancers, are well documented and described [1–4].

However, there are still patients who are prone to higher degrees of acute and late RT toxicity. Risk factors include systemic therapy, obesity, smoking, and the volume of the planning target volume (PTV) [1–3].

However, the density of the breast tissue, the texture of the mammary gland tissue, and biomechanical and pathophysiological properties are poorly investigated so far [4].

Systematic studies on representative cohorts are not available, as data collection is very time-consuming and the necessary data are not acquired in clinical routine practice.

By using a surrogate parameter for the density of the breast tissue, we examine the influence of the tissue’s composition to derive aspects for predicting toxicity like breast edema (BE) and skin toxicity in patients who received breast RT.

## Methods

The collective of breast cancer patients and the treatments received have been described in detail elsewhere [5].

In short, the clinical courses of patients treated at our RT center in Westerstede between 2011 and 2021, Lower Saxony, Germany, were retrospectively analyzed. All patients gave consent for using their data in anonymized retrospective analyses. The data processing and evaluation adhered to the guidelines of the Ethics Committee of Hanover Medical School, Germany, No. 10516_BO_K_2022.

**Fig. 1:**
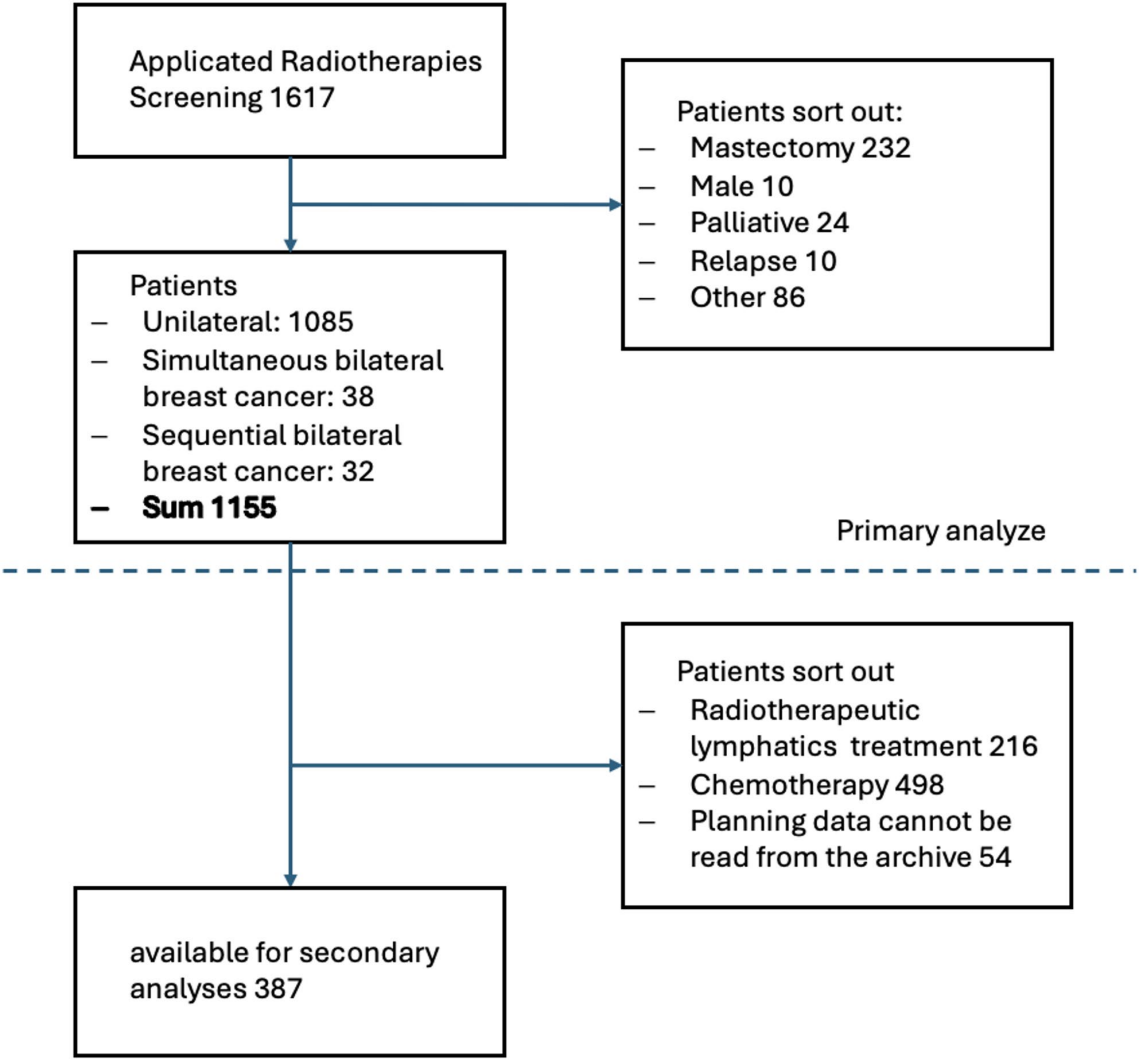
Screening and selection

Inclusion criteria for this analysis were “whole breast RT with or without boost RT” after breast conserving surgery with curative intention. Furthermore, to exclude identified risk factors for the development of post-RT BE, all patients who had received RT of the lymphatic drainage or chemotherapy were excluded, as shown in Fig. 1 [5]. Out of the collective, 387 patients were included in this retrospective analysis.

Patients’ characteristics were documented using the internal patient files. Besides anamnestic, lifestyle, clinical, and RT planning parameters (such as volumes of the primary target volumes (PTV) for “breast” and “boost”), we gave special attention to documentation, grading, and validation of RT-associated toxicity. Skin toxicity was assessed according to “common toxicity criteria 2.0 (CTCAE) [6] weekly during the course of the RT and at 3, 12, 36, and 60 months post-RT, in the event of clinical problems or therapy-related toxicity, with more frequent follow-ups as required. BE was considered therapy-related if it occurred during the structured follow-up and affected the treated breast. Post-RT BE were graded according to the “WST BE classification”, which takes therapeutic interventions into account [5]. In short, BE grade I is defined as “lymphatic drainage performed by the patient”, grade II as “professional lymphatic drainage”, and grade III as “surgical intervention”.

As a surrogate for “breast density”, all RT-planning CT scans were analysed by two investigators (MRO, RMH) using the image display software CHILI v4 (nexus / chili, Heidelberg, Germany).

In the transverse section, the area of the nipple of the affected breast (or, in case of surgical resection, of the contralateral nipple) was examined. In this region, a polygon structure was drawn to include the breast tissue by excluding the skin and subcutaneous tissue, edema, ribs, and pectoral muscles. Furthermore, surgical clips had to be excluded, as well as artifacts from positioning devices (especially in the prone treatment position). The mean values, standard deviations, min, and max of the Hounsfield units (HU) in this “breast structure” were documented.

We divided the patients into groups according to their mean density. A density of > -59 Hounsfield Units (HU) was regarded as glandular tissue and < -59 HU as fatty tissue according to Fogliata et al. [7, 8]. A separate toxicity analysis was performed.

### Statistical analysis

A general descriptive statistical analysis is performed on the selected study group.

The collective was divided into sub-groups with acute skin toxicity of at least grade II and BE of at least grade II. The reference group consisted of patients with no or grade I toxicity.

To compare two ordinal characteristics, the Chi-square test was employed. For the analysis of metric values, an unpaired t-test was used. Where data were not normally distributed, non-parametric t-tests were used. These inferential statistical tests were initially conducted to identify variables with statistical significance. Variables that demonstrated statistical relevance were subsequently included in a multivariable regression analysis in order to determine independent predictors.

A p-value of less than 0.05 was regarded as indicative of statistical significance. All statistical analyses were performed using Prism^®^ 10.4 for Mac and Windows (GraphPad Software Inc., San Diego, CA, USA).

## Results

The analysis included 387 patients from the collective reported in a previous publication who received RT exclusively to the breast.

A boost was applied in 265 patients, and 122 received a homogenous dose distribution to the entire breast. 31 boost applications were performed sequentially after whole breast RT. Most treatments were normofractionated with a dose of 1.8 Gy to 2 Gy. Only 136 received hypofractionated RT. Details are reported in Table 1.

**Table 1:** Descriptive parameters of the collective; ^+^excluded patients without Boost

Age	*All patients (n = 387)*
	59.96 +/- 11.23
*Size*	1.67 +/- 0.06
*Weight*	76.43 +/- 15.92
*Side (left/right) [n]*	198/188
*ypT0/ypTis/pTis*	31
*ypT1/pT1*	279
*ypT2/pT2*	74
*pT3*	3
*PTV Breast Volume [cm* ^*3*^ *]*	1004 +/- 457.6
*Mean Density*	-80.86 +/- 20.89
*PTV Boost Volume [cm* ^*3*^ *]* ^*+*^	69.89 +/- 43.37
*No Boost/SEB/SIB [n]*	122/31/234
*Hypo/Normo-fractionation [n]*	136/251

Higher incidence of skin toxicity grade II was separately proven and was significantly higher (“no boost” skin toxicity I/II 96/13 “with boost” skin toxicity I/II 173/76; *p* < 0.05). The odds ratio was 3.021 (95% CI 1.281 to 6.840, *p* < 0.05) for skin toxicity.

No association was observed between boost radiation vs. no boost radiation and the risk of BE II occurrence in our cohort. Details are reported in Table 2 and Table S1* in the supplement*. Furthermore, patients who received normofractionated RT were overrepresented in the group with skin toxicity grade II.

**Table 2:** Comparison of patients with Grade I/0 and Grade II toxicity; *unpaired t-test, **Fisher’s exact-test +without Boost excluded

Age	Skin Tox 0/ I *(n = 298)*	Skin Tox II *(n = 89)*	Different?
	60.67 +/- 11.66	57.57 +/- 9.33	*p* < 0.05*
*Mean Density*	-79.79 +/- 21.29	-84.48 +/- 19.19	*p* = 0.06*
*PTV Breast Volume [cm* ^*3*^ *]*	933.5 +/- 391	1242 + 573.1	*p* < 0.05*
*PTV Boost Volume* ^*+*^ *[cm* ^*3*^ *]*	42.41 +/- 44.10	66.91 +/- 56.99	p = < 0.05*
*No Boost/Boost*	109/189	13/76	*p* < 0.05**
*SIB/SEB [n]*	175/14	59/17	*p* < 0.05**
*Hypo/Normo-fractionation [n]*	132/167	6/83	*p* < 0.05**
	***Edema 0/I*** ***(n = 360)***	***Edema II*** ***(n = 27)***	
*Age*	60.03 +/-11.43	58.93 +/- 9.79	*p* = 0.62*
*Mean Density*	-80.79 +/- 20.70	-81.78 +/-23.79	*p* = 0.81*
*PTV Breast Volume [cm* ^*3*^ *]*	985.6 +/- 444.1	1255 +/-592.9	*p* < 0.05*
*PTV Boost Volume* ^*+*^ *[cm* ^*3*^ *]*	46.69 + 47.46	66.07 +/- 57.71	*p* < 0.05*
*No Boost/Boost*	114/246	8/19	*p* > 0.99**
*SIB/SEB [n]*	218/28	16/3	*p* = 0.47**
*Hypo/Normo-fractionation [n]*	129/221	9/18	*p* = 0.84**

**Fig. 2:**
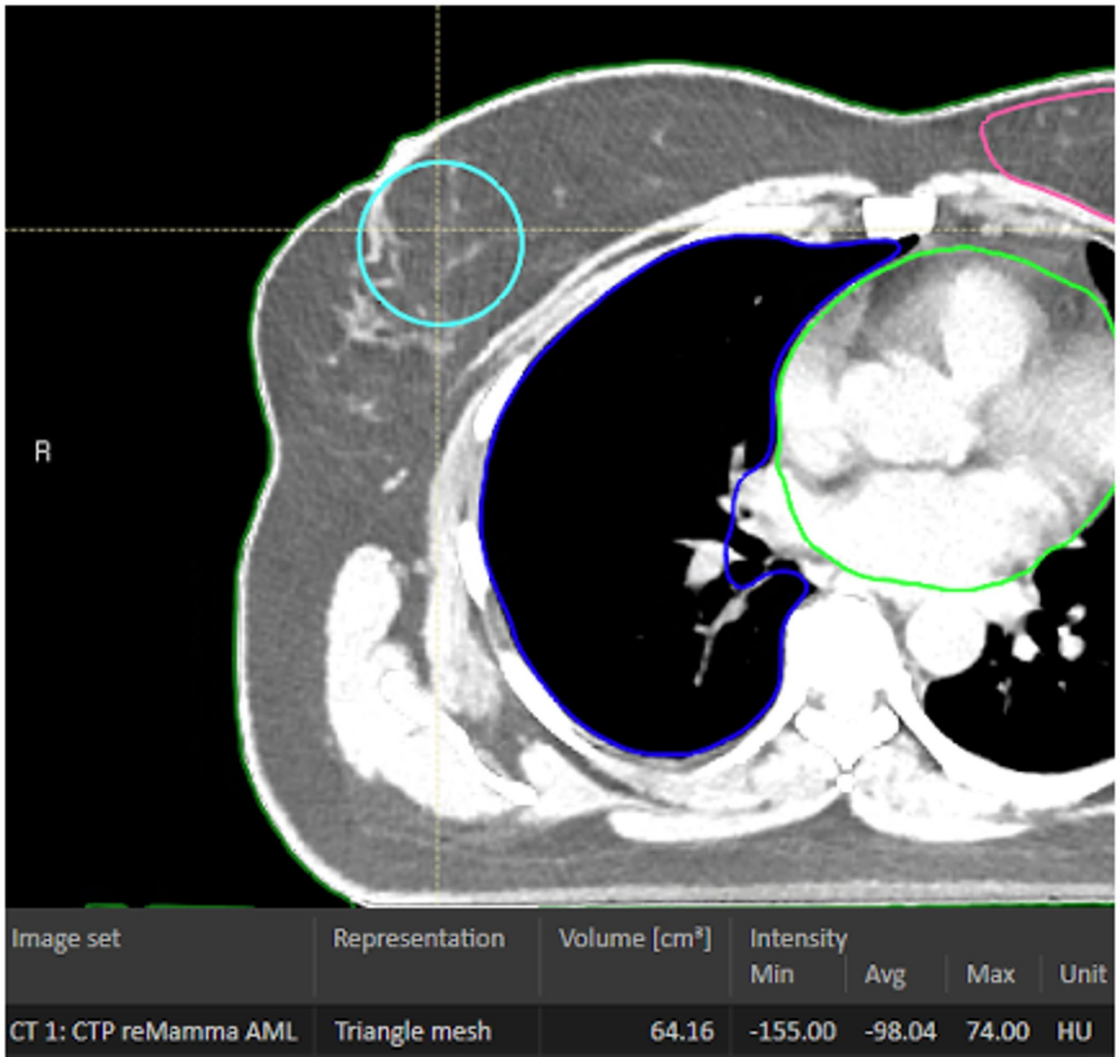
Example of the application of the surrogate maker. The structure is shaped to avoid contact with skin, edema, or bone when necessary, illustrating its ability to adapt to surrounding anatomical constraints

However, for the mean density of the breast tissue, we could not observe any difference between any group (Fig. 2).

Between the density-dependent sub-groups (>-59 HU and <-59 HU), 81 patients reported skin toxicity grade II in the <- 59 HU group, which was statistically significant (*p* < 0.05) (Table 3). Furthermore, there was no significant difference in the number of patients who received normofractionated therapy between the groups. About BE, we could not observe a higher prevalence in any group.

**Table 3:** Comparison of the patients with densities above and below − 59 HU; *unpaired t-test, **Fischer’s exact-test

Hypo/Normofractionation [*n*]	Mean Density >-59 HU (*n* = 55)	Mean Density <-59 HU (*n* = 327)	
	22/33	104/212	*P* = 0.35**
Mean Density	-41.49 +/- 15.49	-88.04 +/- 11.9	*p* < 0.05*
Mean PTV Breast Volume	618 +/- 242.70	1074.0 +/-451.7	*p* < 0.05*
Skin I	49	219	*p* < 0.05**
Skin II	6	81	*p* < 0.05**
Edema grade I	6	69	*p* = 0.10**
Edema grade II	5	21	*p* = 0.58**

**Fig. 3:**
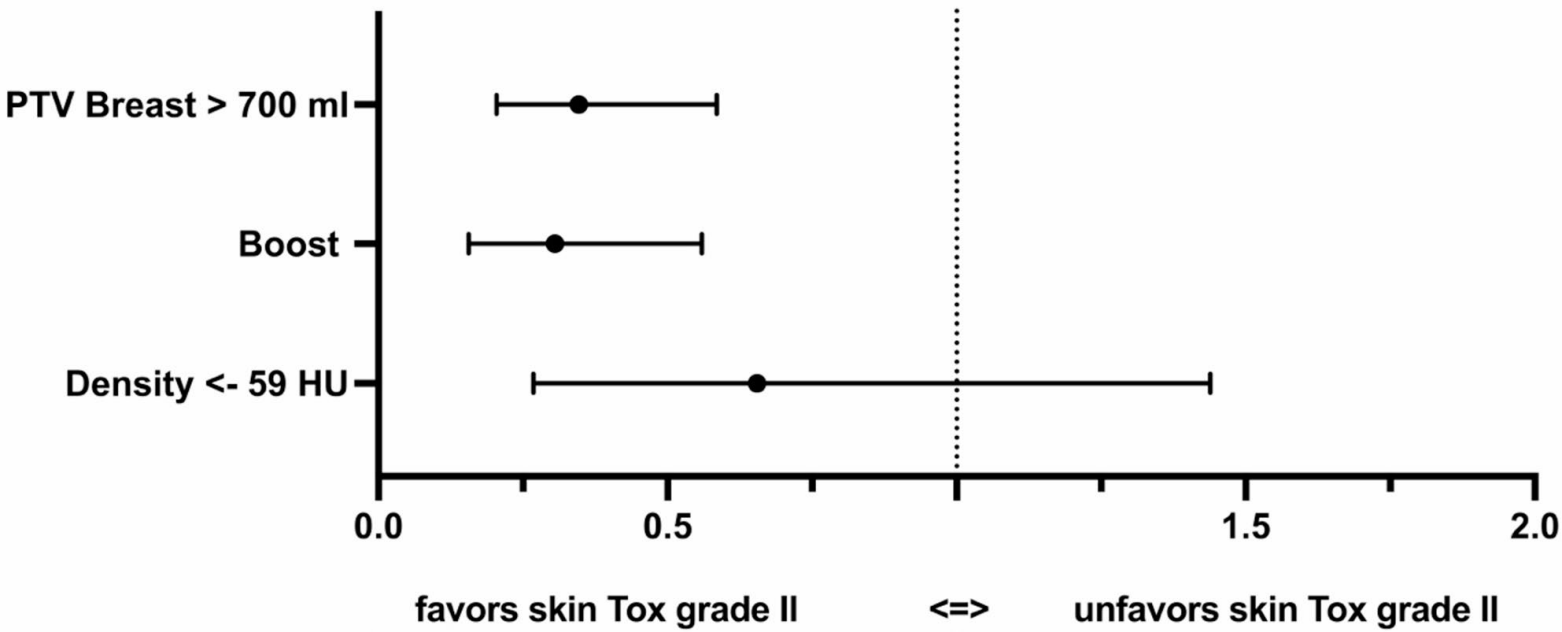
Forest-Plot plot of the mulitivariable analysis of the influence on skin toxicity

The multivariable regression model showed a robust influence of a PTV > 700 mL and the application of a boost as risk factors for skin toxicity grade II. No significant impact of the surrogate marker for breast density was seen in the regression model (Fig. 3).

(ROC Area = 0.69, 95% confidence interval 0.62 to 0.74, *p* < 0.05) (and additional ROC curve available in the Supplemental Figure SI).

However, for BE no significance was demonstrated in the multivariable regression model (Supplemental Figure SII).

## Discussion

The data demonstrates an association between an increase of the volume of the irradiated breast and the risk of developing grade II skin toxicity and/or BE. The risk of developing grade II toxicity depends not only on the PTV breast volume but also on the PTV boost volume. Additionally, for a HU < -59 (our surrogate marker for breast density) we showed a higher risk of developing grade II toxicity of the skin.

Volume-dependent toxicity is well described in the literature for most of the organs at risk during and after RT. The QUANTEC data describe cardiac toxicity following RT of the breast, but there are no systematic descriptions of volume-dependent toxicity of the skin, especially with regards to fatty tissue or mammary glands in the publication [9, 10].

Our data show a relation between the volumes and a risk of developing grade II skin toxicity or grade II breast BE.

Similar to already published data, no increase of the risk for BE can be observed between the hypo- and normofractionated groups in this cohort [5] which compares to the reported BE incidence in the literature [11]. We excluded all patients with chemotherapy and lymphatic RT due to a higher risk of secondary BE in these groups [12]. Resulting from this, we only have the whole breast RT as a main factor for the induction of BE.

However, one aspect that we cannot investigate further, but which also influences the risk of BE, is the type and quality of the surgical intervention [13]. Here too, poorer tissue alimentation can increase the complication rate. The risk of BE is mutually reinforced by RT and surgery.

In our data we assessed a significant association between a higher radiation volume and the risk for developing a BE grade II. We suggest that this effect is not only valid for the PTV breast volume, but also valid for the boost volume. With regards to skin effects, we reported an association between the treatment volume and the development of skin toxicity grade II. This is coherent with the available reports in the literature [1]. Our data also show an association for the volume of the boost. The sequence of the boost application has an influence for the skin toxicity, too, as a sequential boost RT may increase skin toxicity [14]. The proportion of patients who received sequential boost therapy in our cohort is significantly higher in the group with grade II skin toxicity. A delay in skin reaction due to the choice of fractionation is described in the literature [15]. However, a critical point in our collective is the overrepresentation of normofractionated therapy in the group of patients with grade II toxicity, as this may cause an underestimation of the actual incidence of skin toxicity. This effect could be reduced by a more consistent follow-up after the end of RT like suggested by Bouziane et al. [16], but this is only possible to a limited extent in clinical routine.

However, we see the main result of our work in relation to the association between glandular density and fat distribution in the breast using the surrogate marker we applied. In estimating the gland-to-fat ratio, biomechanical estimates in relation to volumes are used [17]. However, some of these are inadequately validated [10]. The surrogate marker we use cannot completely compensate for these weaknesses, but it is nevertheless a useful option for assessing the condition of breast tissue. It is reproducible, and the required computed tomography is already available for RT planning. The mean HU values are read out using standard planning software.

Patients with HU values of <- 59 and thus increased fatty tissue are associated with a significantly higher risk of developing skin toxicity grade II in the inferential analyses. Effects of inhomogeneous dose distribution may lead to misestimating [7] so that overdosing can increase skin toxicity [18]. But these effects are not described as clinically significant [7].

A translational study from 2011 showed a correlation between a higher fat content in the breast and an increased inflammatory activity [19]. Thus, this could also be the reason for the observed correlation between the skin tox grade II and the higher number of fatty tissue of the breast in some patients. The correlation observed in this cohort between grade II skin toxicity and a bigger ratio of fatty tissue in the breast may, therefore, be attributable to the associated pro-inflammatory microenvironment in these patients.

Larger breast volume is also associated with an increase in the fat content of the breast, which may cause more side effects [20]. This could also put obese patients at a higher risk as, both the breast volume and the ratio of fatty tissue in the breast are often increased.

Given the lack of effect of the surrogate parameter in the multivariable model, the absolute volume alone must initially be considered to have the greater impact on toxicity. The available knowledge about breast density and its influence on various physiological and pathophysiological aspects is very limited, especially regarding the influence of glandular density on the side effects of RT [4]. Breast density is repeatedly cited as a risk factor for the development of breast cancer [21].

In previous retrospective analyses, analyses of the composition of breast gland adipose tissue for outcome prediction after radiotherapy did not provide any findings beyond additional biological insights [22]. These findings also relate only to oncology-specific topics. There are no structured studies on toxicity or the influence of mammary gland composition and its effect on radiogenic toxicity.

The regenerative capacity and the tissues composition of a breast’s varying composition can possible influence the toxicity risk and be stratified in advance. A study from 2026, which was first addressed in the literature by Jaikuna et al., coincides with our findings on the relationships between volume, density and the risk of toxicity [23].

In this patient group adapting the treatment strategy and plan could reduce the risk of toxicity or at least toxicity > grade I. A large proportion of the patients would fall within the indication range for partial breast RT [24]. Sufficient evidence of oncological safety is now available based on 10-year data [25]. Even with altered toxicity, intraoperative radiation therapy is a valid alternative in selected patient groups [26]. For patients who do not meet the criteria for partial breast RT, a dose escalation in line according to the IMPORT-HIGH study can be considered, at least for whole breast RT [27].

The retrospective nature of our dataset allows only a predominantly descriptive interpretation of the presented results. Moreover, the surrogate marker used to estimate mammary gland composition requires further validation, ideally through targeted comparison with the absolute dimensions of glandular tissue in relation to total breast volume. In this context, our assumption cannot be adequately represented in the multivariate model.

Also the surrogate parameter selected in this study has inherent limitations regarding its validity. A full autosegmentation of the entire mammary gland within the PTV would provide a more representative reference. Although we aimed to minimise intraobserver variability by involving two independent investigators, some degree of variability cannot be fully excluded.

The predominance of normofractionated treatment regimens—already discussed above—likely contributes substantially to the high rate of grade II skin toxicity observed in our cohort. It should be emphasized that hypofractionated concepts ought to represent the standard of care in this specific indication of adjuvant breast radiotherapy. Prospective studies are therefore necessary to verify whether the conclusions drawn here can be analogously applied to hypofractionated treatment approaches.

In addition, the surgical technique used in breast-conserving therapy may represent an additional influencing factor on tissue composition and subsequent radiation effects. This parameter should be systematically evaluated in future studies to better understand its potential impact on treatment outcomes.

The existing correlation between the surrogate marker for breast tissue and toxicity indicates that this aspect needs to be mapped more precisely to characterize the influence on toxicity under breast RT. However, this requires a prospective study design and an adjustment of the methodology for measuring breast density. Treatment volume-adapted therapy in patients with large breast volumes may be a useful option to prevent higher toxicity.

## Conclusion

Inferential statistics show a correlation between breast volume, boost application and breast density with skin toxicity and the risk of BE. The multivariable model can only support this correlation to a limited extent, so prospective studies are necessary to further characterise it. The observed correlation suggests breast tissue characteristics should be mapped more precisely; this requires prospective studies and refined breast density measurement methods. Adapting therapy to treatment volume may help reduce toxicity in patients with large breasts.

## Supplementary Information

Below is the link to the electronic supplementary material.

Supplementary Material 1

Supplementary Material 2

## Data Availability

The data presented in this study are available on request from the corresponding author.
