# Correction: Association between radiation volume and breast density for skin toxicity and breast edema after radiotherapy in breast conserving therapy of breast cancer

**DOI:** 10.1186/s13014-026-02849-2

**Published:** 2026-06-17

**Authors:** Mathias Sonnhoff, Melsa Oyur, Jan-Nicklas Becker, Robert Michael Hermann, Cedric Oliver Carl, Mirko Nitsche, Hans Christiansen, Robert Maximilian Blach

**Affiliations:** 1 Department of Radiotherapy, Hannover Medical School, Carl-Neuberg- Straße 1, 30625 Hannover, Germany; 2 IMAGINE-Niedersachsen, Carl-Neuberg-Str. 1, 30625 Hannover, Germany; 3 Radiotherapy NORD Bremen and Westerstede, Bremen, Germany; 4 Department of Radiation Oncology, University Medical Center Schleswig-Holstein, Kiel, Germany

**Correction: Radiation Oncology (2026) 21:51**

10.1186/s13014-026-02833-w

After publication of the article, it was brought to our attention that the first name and the family name of all authors were swapped. The original publication has been corrected.

