# Supplementary material for: Association between radiation volume and breast density for skin toxicity and breast edema after radiotherapy in breast conserving therapy of breast cancer
Source: Radiat Oncol. 2026 Mar 28;21:51. doi: 10.1186/s13014-026-02833-w (PMC13063576; doi:10.1186/s13014-026-02833-w)
Supplement: Supplementary file 2 — Supplementary Material 2 [file 13014_2026_2833_MOESM2_ESM.docx]

## Supplement material:

TableS I: *unpaired t-test, **Fischer’s exact-test

|  | *No Boost  (n= 122)* | *Boost (n=265)* |  |
| --- | --- | --- | --- |
| *Age* | 66.56 +/- 9.72 | 56.92 +/- 10.56 | p<0.05* |
| *Side (left/right) [n]* | 64/58 | 135/130 | p = 0.82** |
| *PTV Breast Volume [cm^3^]* | 996.5 +/- 439.7 | 1008 +/- 466.4 | p= 0.82* |
| *Mean Density* | -83.21 +/- 19.11 | -79.77 +/- 21.62 | p= 0.13* |
| *PTV Boost Volume [cm^3^]* | n. a. | 69.54 +/-43.93 | n. a. |
| *SIB/SEB [n]* | n. a. | 234/31 | n. a. |
| *Hypo/Normofractionation [n]* | 77/45 | 59/206 | p <0.05** |
| Skin Toxicity I | 96 | 173 | p= 0.42* |
| Skin Toxicity II | 13 | 76 | p<0.05* |
| Edema grade I | 19 | 55 | p>0.99* |
| Edema grade II | 9 | 18 | p=0.57* |

TableS III:

|  | Mittelwert >-80 | Mittelwert <-80 |  |
| --- | --- | --- | --- |
|  | 14 | 15 | p= 0.21 |
| *Hypo/Normofractionation [n]* |  |  |  |
| Skin I | 101 | 167 | p= 0.32 |
| Skin II | 27 | 61 |  |
| Edema any | 29 | 72 | p = 0.055 |
| Edema Grade II | 8 | 18 | p = 0.84 |

## multivariable regression Skin Toxicity grade II

| Variable | Odds ration | 95%-CI | p-valuve |
| --- | --- | --- | --- |
| Boost | 0.3049 | 0.1561 to 0.5594 | <0.05 |
| Densitiy < -59 HU | 0.6550 | 0.2681 to 14.39 | 0.32 |
| PTV-Breast | 0.3467 | 0.2041 to 0.5852 | <0.05 |


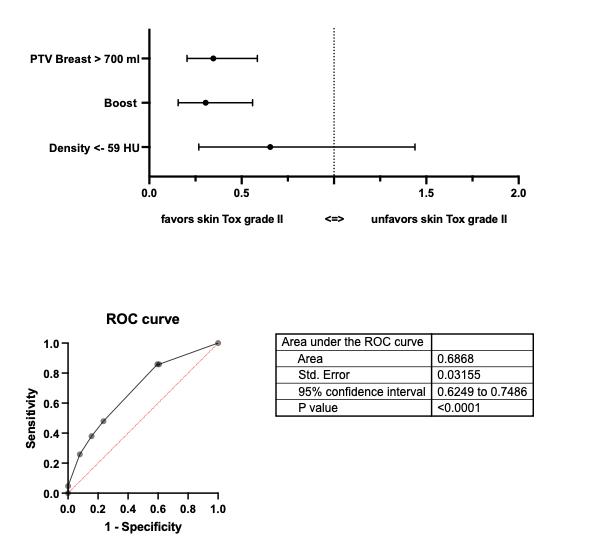


figureS I: ROC-model,

| Variable | Odds ration | 95%-CI | p-valuve |
| --- | --- | --- | --- |
| Boost | 1.022 | 0.04419 to 2.570 | 0.96 |
| Densitiy < -59 HU | 0.6874 | 00.2243 to 2.563 | 0.54 |
| PTV-Breast | 2.332 | 0.9819 to 5.609 | <0.05 |


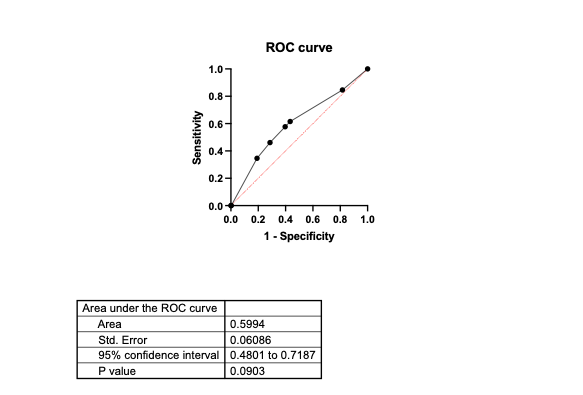


figureS II: multivariable model for edema no significant result,
