# Supplementary material for: Association between radiation volume and breast density for skin toxicity and breast edema after radiotherapy in breast conserving therapy of breast cancer
Source: Radiat Oncol. 2026 Mar 28;21:51. doi: 10.1186/s13014-026-02833-w (PMC13063576; doi:10.1186/s13014-026-02833-w)
Supplement: Supplementary file 2 [file 13014_2026_2833_MOESM1_ESM.docx]

Odds ratios	Variable	Estimate	95% CI (profile likelihood)
β0	Intercept	0.07133	0.01816 to 0.2146
β1	Boost ja/nein[1]	1.022	0.4419 to 2.570
β2	<-59[1]	0.6874	0.2243 to 2.563
β3	350-500< D und mehr	2.332	0.9819 to 5.609

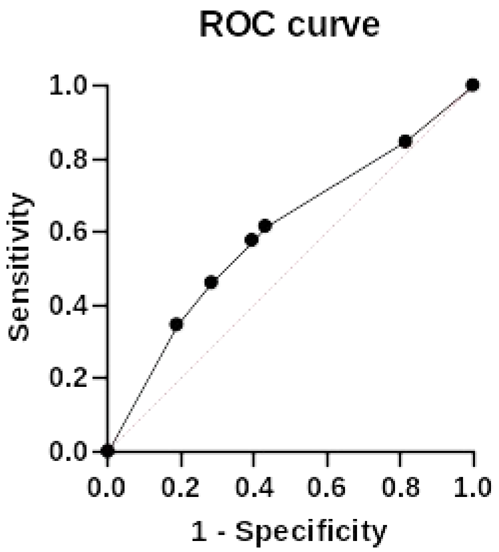

Area under the ROC curve	
Area	0.5994
Std. Error	0.06086
95% confidence interval	0.4801 to 0.7187
P value	0.0903
